# Cross-interface persistent memory for large language model-assisted radiology research workflows: a Claude-based proof-of-concept technical development

**DOI:** 10.3389/fradi.2026.1890122

**Published:** 2026-08-17

**Authors:** Miguel Angarita, Lina M. Acosta Buitrago, Juan Manuel Pérez

**Affiliations:** 1 LaCardio — Fundación Cardioinfantil, Bogotá, Colombia; 2 Universidad del Rosario, Bogotá, Colombia

**Keywords:** artificial intelligence, clinical informatics, large language models, low-resource settings, open-weights models, persistent memory, proof of concept, radiology research workflow

## Abstract

**Background and purpose:**

Large language model (LLM)-assisted radiology research and tool-development work is hampered by a practical form of memory loss we term cross-interface fragmentation: project context built in a web-based conversational interface is not available in a local coding-agent environment, and neither environment retains state across sessions. This forces researchers to manually reconstruct context at every session start and every interface transition. This technical development describes the design and a single-user, proof-of-concept functional demonstration of a Claude-based persistent-memory system intended to reduce this fragmentation for the software-engineering and research-organization layer of radiology AI projects. The system is not a clinical tool and does not process patient data.

**Materials and methods:**

We implemented an end-to-end pipeline integrating a custom web application (Created.app/Supabase), a self-hosted n8n automation server, the Claude API (Anthropic), GitHub version control, and the Obsidian knowledge-management application. A web session is summarized by the Claude API into six structured fields (summary, decisions, pending tasks, artifacts, errors, continuation point) and written as a per-project MEMORY.md file to GitHub; a complementary CLAUDE.md file holds accumulated technical context for the local coding agent. The study design is a single-user, single-operator proof-of-concept engineering validation across three concurrent development projects; no efficacy, usability, or clinical-outcome claim is made.

**Results:**

In functional testing, the pipeline transferred project context between the web and local-coding environments without manual copy-paste of session content. A representative 558-token session (*n* = 1 observation) was processed end-to-end with complete six-field extraction; create and edit operations against the GitHub Contents API succeeded across five consecutive runs without overwrite errors. On a fresh launch, the local coding agent read the memory files and reported project identity, prior technical decisions, and pending tasks without user-provided context. Reported timings are single-run observations, not benchmarked measurements.

**Conclusion:**

A persistent, cross-interface memory layer for LLM-assisted radiology research workflows is technically feasible with currently available components. As a proof of concept it is explicitly limited: single user, Claude-based, text-only, without quantitative extraction-accuracy benchmarking, and dependent on commercial APIs whose cost scales with use (fixed infrastructure plus variable per-session API cost). We discuss a vendor-neutral, open-weights configuration as the recommended direction for cost- and dependency-sensitive settings such as Latin America, and we report that the system has been used in practice to help organize the development of a separate radiology report quality-control tool.

## 1 Introduction

Large language model (LLM)-based tools have shown substantial promise in radiology, including report drafting, structured reporting, differential-diagnosis support, and workflow optimization (1, 2). A growing share of this work is not clinical interpretation but development work: radiologists and informatics researchers increasingly use LLMs to design protocols, reason about study methodology, write and debug code, and build radiology-specific AI tools. This development work spans weeks to months and accumulates substantial project state — decisions, rationales, configurations, and pending tasks.

A practical limitation constrains this development work: LLMs are stateless across sessions, and the interfaces through which they are accessed are mutually isolated. Researchers commonly work in two structurally different environments — web-based conversational interfaces, convenient for reasoning and discussion but ephemeral, and local coding-agent environments, persistent on disk but isolated from the web conversation. Context built in one environment is not available in the other. We refer to this combined problem — statelessness within an environment plus isolation between environments — as cross-interface fragmentation. Its practical cost is repeated manual reconstruction of accumulated context at every session start and every interface transition.

This problem is increasingly recognized, and several commercial LLM platforms now offer partial solutions, including conversation-scoped and account-scoped memory features and project or workspace containers. These systems are valuable but share two limitations relevant here: they are generally confined to a single vendor's environment and do not bridge a web chat interface and an independent local coding agent; and they expose memory as an opaque, vendor-controlled store rather than as a transparent, user-owned, version-controlled artifact. The contribution of the present work is therefore deliberately modest and specific: a transparent, file-based memory layer that (i) bridges a web conversational interface and a local coding agent, (ii) is owned and versioned by the user in plain Markdown, and (iii) is designed so the extraction model can be swapped, including for a self-hosted open-weights model.

To avoid the terminological ambiguity noted in review, Table 1 defines the terms used throughout this manuscript. The unit of persistence we target is the project, not the individual conversation; the system is concerned with project continuity for development work — not with conversation continuity and not with clinical-content memory.

**Table 1:** Definitions of memory-related terms as used in this manuscript. The unit of persistence is the project, not the conversation.

Term	Definition as used in this manuscript
Context window	The bounded amount of text an LLM can attend to within a single inference call.
Session	A single continuous interaction with an LLM in one environment (one web conversation, or one local coding-agent run).
Conversation	The web-based dialogue specifically; a subtype of session. Not the unit of persistence in this work.
Project	A multi-week development effort spanning many sessions and both environments. The unit of persistence targeted here.
Session Memory	Context from one specific session (what was discussed or decided in that interaction).
Project Memory	The accumulated longitudinal record of decisions, artifacts and pending tasks across all sessions of a project (stored in MEMORY.md).
Technical Memory	Accumulated implementation context for the local coding agent: architecture, APIs, code decisions (stored in CLAUDE.md).
Persistent memory	Project + Technical Memory written to durable, version-controlled files that survive across sessions, devices and environments.

Inspired by the LLM Wiki pattern described by Karpathy in an April 2026 GitHub Gist (5) — a model-maintained, plain-text knowledge file that a coding agent reads at session start and updates over time — we extend that single-environment, code-only concept to additionally capture web-conversation context and synchronize it across environments. We describe the architecture, implementation, and a single-user proof-of-concept functional demonstration. We make no efficacy, usability, scalability, or clinical claims; these are out of scope and noted as future work where relevant.

## 2 Materials and methods

### 2.1 Study design

This is a single-user, single-operator proof-of-concept engineering validation. All implementation and testing were performed by the first author across three concurrent software-development projects. The objective was to determine whether project context could be transferred automatically between a web conversational interface and a local coding agent, without manual copy-paste of session content, using only currently available components. No comparison group, baseline, multi-user evaluation, or statistical hypothesis test was performed; the design supports feasibility conclusions only.

### 2.2 Scope and data handling

The system stores software-development and research-organization context only: session summaries, engineering decisions, pending development tasks, code artifacts, errors, and a continuation point, plus accumulated technical notes for the coding agent. It does not store, process, or transmit patient data, imaging data, clinical reports, or any identifiable personal information. The three development projects used as the test environment are software products; none contains patient data. This scope is a deliberate design boundary, not an omission, and it is the basis for the data-governance discussion in Section 4.6.

### 2.3 Conceptual memory model

**Figure 1:**
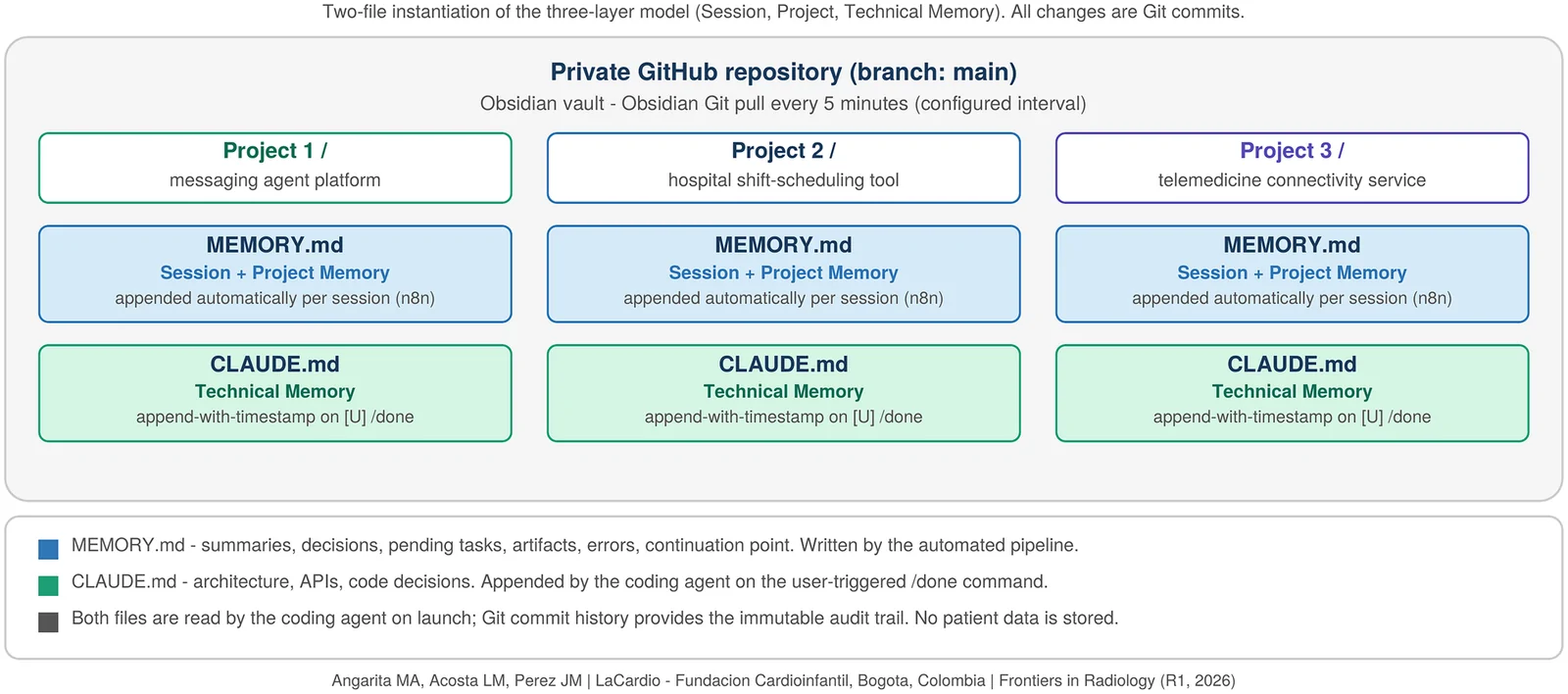
Vault structure: two memory files per project. Vault structure and two-file instantiation of the three-layer model. Each project subdirectory contains MEMORY.md (Session + Project Memory, appended automatically per submitted session) and CLAUDE.md (Technical Memory, updated by the coding agent on/done). Both are read on coding-agent launch; both are version-controlled in Git.

Memory is organized into three conceptual layers, instantiated as two physical files per project (Figure 1):

- Session Memory — context from one specific interaction.
- Project Memory — the longitudinal record of decisions, artifacts and pending tasks across all sessions, stored in MEMORY.md and appended (never overwritten) per session.
- Technical Memory — implementation context for the coding agent (architecture, APIs, code decisions), stored in CLAUDE.md.

Session and Project Memory are written to MEMORY.md; Technical Memory is written to CLAUDE.md. We refer to these two files collectively as the project's persistent-memory set. We note explicitly that this is a two-file instantiation of a three-layer conceptual model, and we have removed stronger framing (“cognitive state layer,” “transformative”) used in the original submission as not warranted by the artifact, which is a set of version-controlled Markdown files.

The unit of memory is the project, not the conversation. Sessions are ephemeral; the project persists across sessions, devices and interfaces.

### 2.4 System architecture

**Figure 2:**
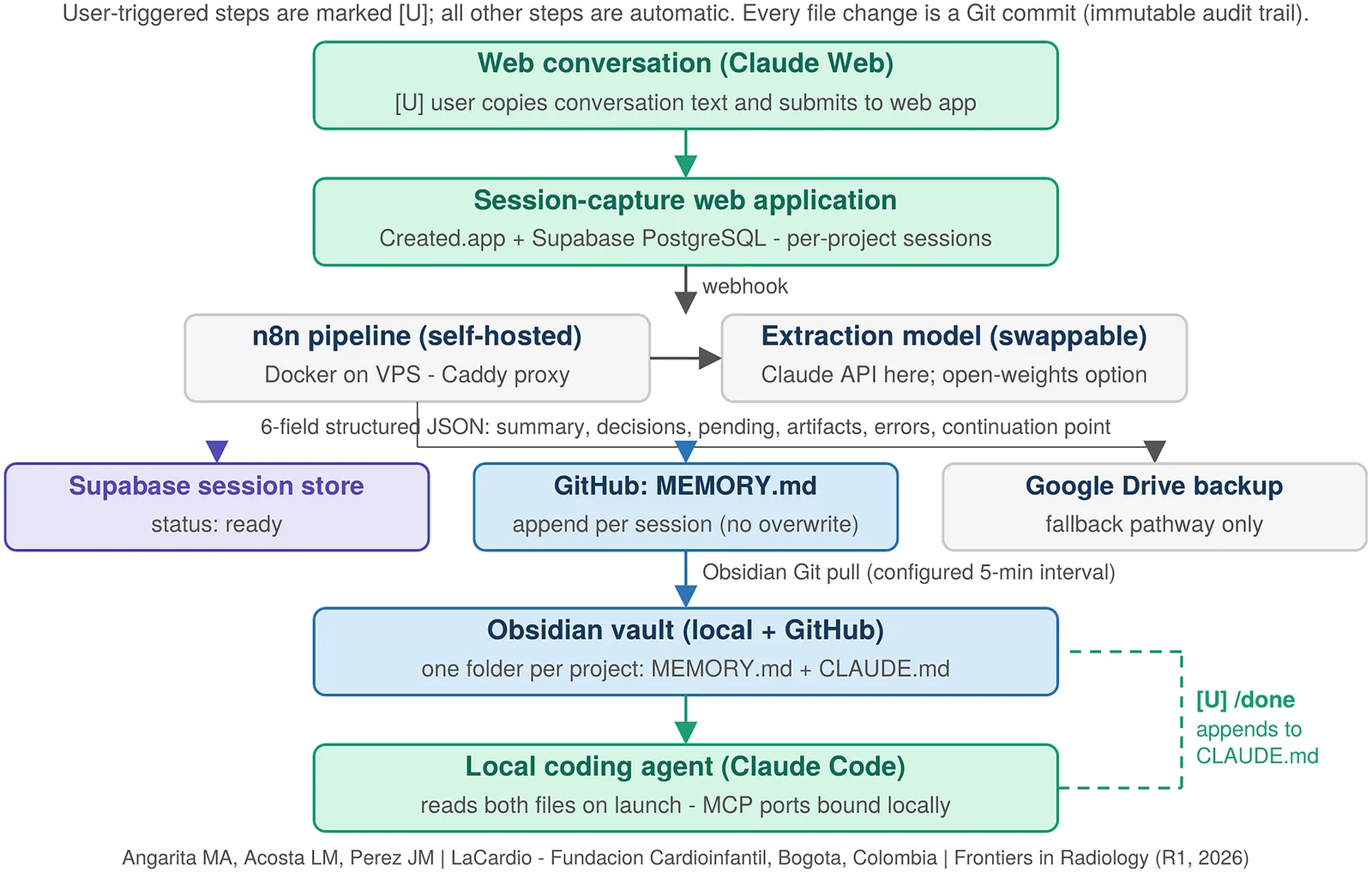
End-to-end data flow of the persistent-memory system. End-to-end data flow. A web conversation is copied into the web application and submitted (a user-triggered step). An n8n webhook receives the text; the Claude API extracts six structured fields; the primary pathway updates the session store and writes/append-updates the project MEMORY.md in GitHub via the Contents API; a fallback pathway backs up raw text to Google Drive. The Obsidian Git plugin pulls every 5 min. The local coding agent reads MEMORY.md and CLAUDE.md on launch. Typing/done updates CLAUDE.md (also user-triggered). All file changes are Git commits, providing an immutable audit trail.

The system operates across three functional layers; Figure 2 is the complete data-flow diagram.

#### 2.4.1 Layer 1 — session capture

A custom web application (Created.app, backed by a Supabase PostgreSQL database) provides per-project session management for three concurrent development projects: a messaging-based agent platform for physicians, a hospital shift-scheduling tool, and a telemedicine connectivity service. To capture a web conversation, the user copies the conversation text into the web application and submits it; we make this manual submission step explicit here because it bears on the “capture mechanism” and “zero manual operations” points raised in review (Sections 2.6 and 4.7). The application stores, per session: project identifier, date and time, raw text, an AI-generated summary, structured arrays for decisions, pending tasks, artifacts and errors, a continuation-point string, and a processing status.

#### 2.4.2 Layer 2 — automated processing pipeline

A self-hosted n8n automation instance (v1.121.0; Docker on a Digital Ocean Ubuntu VPS, behind a Caddy reverse proxy) executes, on submission:

1. Webhook reception of the session payload (project, date, time, raw text).
2. JavaScript preprocessing to assemble the Claude API request with a fixed extraction prompt (the prompt template and example JSON are provided in the replication repository; see Data Availability).
3. Claude API call (model: claude-sonnet-4, accessed April 2026 via the Anthropic Messages API) requesting structured JSON with six fields: summary, decisions, pending tasks, artifacts, errors, continuation point. The model identifier is not pinned to a specific version; any maintained deployment must accommodate model substitution as identifiers change over time.
4. JSON parse and conditional branch: on success, route to the primary pathway; on parse failure, route to a fallback pathway.
5. Primary pathway: update session status via the application API; write or update the project MEMORY.md via the GitHub Contents API (create if absent, otherwise fetch the current SHA and edit, appending the new session below prior content).
6. Fallback pathway: persist partial data via the application API and upload the raw session text to Google Drive as a backup.

**Figure 3:**
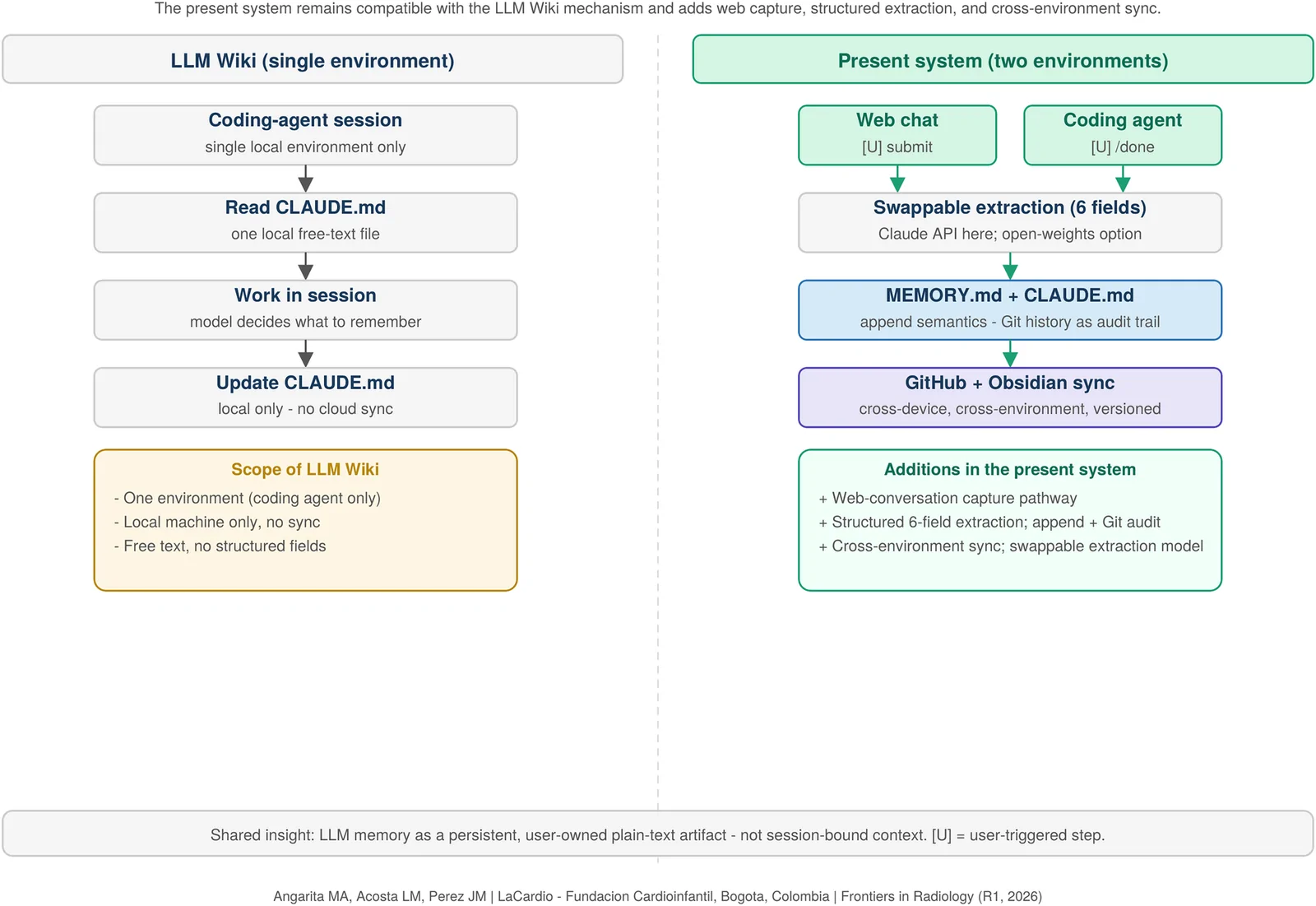
Comparison with the LLM wiki pattern (karpathy, 2026). The LLM Wiki operates in a single local environment with one free-text file, addressing within-environment statelessness. The present system adds web-conversation capture, structured six-field extraction, cross-environment synchronization via GitHub, and a swappable (including open-weights) extraction step, while remaining compatible with the LLM Wiki mechanism for the coding-agent file.

Layer 2: The comparison is illustrated in Figure 3.

#### 2.4.3 Layer 3 — local knowledge vault

A private GitHub repository (branch main) is the cloud store for an Obsidian vault, organized one subdirectory per project, each holding MEMORY.md and CLAUDE.md. The Obsidian Git plugin is configured to pull every 5 min. The Claude Code MCP plugin exposes the vault to the local coding agent over WebSocket (port 49201) and HTTP/SSE (port 22360) on the operator's machine, so the agent can read the files on launch from a project subdirectory. Network-exposure implications of these ports are addressed in the security discussion (Section 4.5).

### 2.5 Dual-file memory and update semantics

Table 2 summarizes the two files. One reviewer correctly noted that overwriting technical memory would destroy the audit trail. In this revision, MEMORY.md is append-only by construction (each session is added below prior content). For CLAUDE.md, which previously was overwritten on update, we now recommend and document an append-with-timestamp policy plus reliance on Git history as an immutable, externally verifiable audit log: because every change is a Git commit, the full revision history of both files is preserved and inspectable regardless of in-file update policy. We discuss the remaining verification gap (extraction is non-deterministic) in Section 4.4.

**Table 2:** The two persistent-memory files per project and their update semantics. All changes are Git commits, providing an external immutable audit trail.

Dimension	MEMORY.md (Session + Project)	CLAUDE.md (Technical)
Written by	n8n automation pipeline	Local coding agent (Claude Code)
Trigger	Each web session submitted	User types/done at session end
Content	Summary, decisions, pending, artifacts, errors, continuation point	Architecture, APIs, code decisions
Environments	Web + local coding agent	Local coding agent only
Update mode	Append per session (no overwrite)	Append-with-timestamp (revised); full history in Git
Audit trail	Git commit history (immutable)	Git commit history (immutable)

### 2.6 What is automatic and what is user-triggered

To correct an overstatement in the original submission (“zero manual operations”), we state the division precisely. User-triggered steps: (a) copying a web conversation into the web application and submitting it; (b) typing/done at the end of a local coding session to update CLAUDE.md. Automatic steps once a session is submitted: extraction, GitHub write, vault synchronization, and reading of memory files by the coding agent on launch. The accurate benefit is therefore not “less manual effort” in absolute terms, but structured, versioned, cross-interface persistence: the information that is transferred is automatically structured into six actionable fields and made available in both environments without copy-paste of raw context. Programmatic capture — routing the conversation directly from the LLM provider to the pipeline without manual pasting — would be feasible if the provider exposes a conversation-export API or webhook, and is a planned extension (Section 4.8).

### 2.7 Infrastructure and cost model

Table 3 corrects the cost description. The fixed infrastructure cost is approximately USD 12/month (the VPS). However, the stack also includes commercial, usage-billed components — the Claude API (per-token) and the Created.app hosting platform — so the system is not “entirely open-source/free-tier,” as the original submission stated. Total cost is therefore fixed cost plus a variable per-session API cost that scales with usage. Under light single-user research use the variable cost is small, but it is non-zero and grows with volume; we make this explicit rather than implying a flat USD 12/month.

**Table 3:** Transparent cost model: one fixed infrastructure cost plus a variable, usage-billed API cost. The original “entirely open-source/free-tier” description is corrected.

Component	Cost type	Notes
Digital Ocean VPS (n8n)	Fixed ∼USD 12/mo	Only fixed recurring cost
Claude API (extraction)	Variable per token	Commercial; scales with session volume
Created.app (web app hosting)	Commercial tier	Not open-source; free tier used here
Supabase/GitHub/Obsidian/Obsidian Git/MCP plugin	Free/open-source	Free-tier or open-source components
Google Drive (fallback backup)	Free tier	Optional fallback pathway only

### 2.8 Functional validation procedure

End-to-end functional testing was performed on April 24, 2026 with a representative 558-token session submitted to one project. We recorded: whether the pipeline executed end-to-end; whether all six fields were populated; whether GitHub create and edit operations succeeded across consecutive runs; and whether the coding agent, launched from the project subdirectory with no user-provided context, reported the known project state. All quantities reported in Results are single-run (*n* = 1) observations from this functional test, except the create/edit check, which was repeated five times. We did not establish ground truth for extraction quality, did not compute precision/recall/F1, and did not test multiple users, long or ambiguous sessions, or failure injection; these are stated as limitations (Section 4.7) and future work. The extraction prompt used in this validation is reproduced in full in the Appendix.

## 3 Results

Results are reported as a functional demonstration, not as performance benchmarks. Single-run timings are labelled as such.

### 3.1 End-to-end execution

The pipeline executed end-to-end on the primary pathway without error. The 558-token session produced a complete six-field extraction (one summary, one decision, four pending tasks, one artifact, zero errors, one continuation point). For this single run, elapsed time from webhook receipt to GitHub write completion was approximately 47 s and session status updated within approximately 45 s (both *n* = 1 observations, not benchmarked).

### 3.2 Memory-file operations

Create and edit operations against the GitHub Contents API succeeded across five consecutive runs. The first run created the project MEMORY.md with all six sections; subsequent runs fetched the current SHA and appended new content below prior content. No overwrite or data-loss error was observed in these five runs.

### 3.3 Vault synchronization

The Obsidian Git plugin pulled updated content from GitHub. With the configured 5-minute polling interval, synchronization occurred within that interval; a manual command-palette sync completed in under approximately 30 s (*n* = 1). We note, per review, that the “under 5 min” figure is the configured polling interval, not an independent latency measurement.

### 3.4 Context retrieval by the coding agent

Launched from a project subdirectory with no user-provided context, the local coding agent read MEMORY.md and CLAUDE.md and reported the project identity and scope, prior architecture and deployment decisions, the pending implementation tasks, and the continuation point, consistent with the known ground-truth project state for that single test. No incorrect item was reported in this single run; we do not generalize from one run to an accuracy claim.

### 3.5 Use in a radiology tool-development context

Beyond the three software projects above, the memory system has been used in practice to help organize the development of a separate radiology-specific tool: an LLM-based system for automatic detection of mechanical errors in radiology reports (for example inverted laterality, empty measurement fields, and findings present in the body but absent from the conclusion). In that work the memory system served its intended role — carrying engineering decisions and pending tasks across web and local-coding sessions during tool development. We report this only to evidence applicability in a genuine radiology development context; the separate tool, its data, its institutional setting, and its performance are outside the scope of this manuscript and are not reported here.

## 4 Discussion

### 4.1 Principal findings

Within the limits of a single-user proof of concept, the demonstration shows that a transparent, file-based memory layer can carry project context automatically between a web conversational interface and a local coding agent, without manual copy-paste of session content, using only currently available components. The contribution is an integration pattern and a transparent, user-owned memory artifact, not a validated method or a clinical system.

### 4.2 Relation to commercial memory features

Commercial chat and coding products increasingly offer memory. The distinguishing properties here are narrow but real: the memory spans two independent environments (a web chat and a separate local coding agent) rather than living inside one vendor's product; it is stored as plain, user-owned, version-controlled Markdown rather than an opaque managed store; and the extraction component is swappable, including for a self-hosted open-weights model (Section 4.3). We do not claim superiority on retrieval quality — which we did not benchmark — only these architectural differences.

### 4.3 Cost and vendor dependence: an open-weights configuration

A reviewer correctly observed that, as originally described, the system depends end-to-end on US-based commercial providers (Anthropic for extraction and the coding agent, GitHub for storage, Google for backup), which is in tension with a sovereignty and low-dependence argument for Latin American settings, and that a pinned model identifier is exposed to vendor lifecycle changes. We accept this and separate two claims that the original submission conflated: low cost and low dependence are not the same property.

The architecture is deliberately model-swappable at the extraction step. A vendor-neutral configuration replaces the hosted extraction API with a self-hosted open-weights model (for example a Llama-, Mistral- or Qwen-class model) run on the same or on-premise infrastructure, and can replace provider-specific file naming with a neutral convention (for example AGENT.md alongside MEMORY.md) and US-hosted storage with self-hosted equivalents (for example a self-hosted Git service and object store). We present this open-weights, self-hostable configuration as the recommended direction where data sovereignty or dependency-minimization matters. We note, consistent with our scope, that in our own related radiology tool-development work the extraction/inference component is an open-weights model rather than a hosted frontier model, which we offer as evidence that the open-weights direction is practical rather than hypothetical; we do not report that tool's results here. We also flag model-deprecation risk explicitly: any pinned model identifier (including claude-sonnet-4) will eventually change, and a maintained deployment must accommodate model substitution.

### 4.4 Clinical-safety considerations and the verification gap

Even though this system does not handle patient data, it is appropriate to address the safety pattern a reviewer raised, because the same architecture could later be applied near clinical work. Memory is produced by a non-deterministic model and is subsequently read by an agent that can act on it. A mis-extracted decision or task could therefore propagate. Two mitigations are relevant. First, the Git commit history provides an immutable, externally inspectable audit trail of every memory change. Second, we recommend a human verification gate — extracted decisions and tasks are confirmed by the user before they are treated as authoritative — for any future use adjacent to clinical work. We are explicit that the present proof of concept does not implement an automated verification gate, and that no autonomous action on clinical content occurs because no clinical content enters the system.

### 4.5 Security considerations

A clinically adjacent deployment would require a threat model; we outline the minimum one and the current posture honestly. The n8n webhook that receives submitted text should be authenticated/signed; self-hosted n8n carries a patching and CVE burden that falls on the operator; credentials (a GitHub write-scoped token, Supabase keys, the Claude API key, and Google OAuth) must be stored in a secrets manager and rotated, and we note that an OAuth token expired during testing, illustrating exactly this lifecycle burden; the MCP ports exposed on the operator's machine (49201, 22360) must be bound to localhost or a private network (for example via a mesh VPN) with appropriate TLS, not exposed publicly; and, because an agent reads MEMORY.md and can act on it, upstream content is a prompt-injection/memory-poisoning vector and submitted content should be treated as untrusted. The present proof of concept does not implement webhook signing or a secrets vault; we list these as required hardening before any non-personal-use deployment, and as future work.

### 4.6 Data governance

The strongest governance control here is scope: the system never ingests patient data or clinical content. It stores only software-engineering and project-management context. Consequently the patient-data questions raised in review — PHI de-identification, cross-border transfer of clinical data, applicable health-data regulation — do not arise for the system as described and tested, because there is no patient data to govern. We make this boundary explicit so the system is not misread as a clinical data pipeline. Should the architecture ever be extended toward clinical content, the de-identification, encryption, access-control, regulatory (including Colombia's Ley 1581/2012) and data-processing-agreement requirements would all apply and would need to be satisfied by design before deployment; that extension is out of scope here.

### 4.7 Limitations

This work has substantial limitations, which we state plainly (1). It is a single-user, single-operator proof of concept; nothing about multi-user or team governance is evaluated (2). It is Claude-based as implemented and tested; cross-platform generality is argued by design (model-swappable extraction) but not demonstrated (3). It is text-only; radiology is inherently multimodal (DICOM, PACS metadata, images), and the system was built for text because its first practical application was a text-report tool — image handling is not implemented (4). Extraction quality was not benchmarked; we report no precision, recall, F1, omission, duplication or hallucination rates, and these should be measured against pre-stated ground truth in future work (5). Reported timings are single-run observations (6). Failure modes (synchronization conflicts, corrupted files, API or GitHub outages, token overflow as memory grows) were not systematically tested (7). Memory files are appended without compression, so scaling behavior over long projects is unaddressed (8). Security hardening (webhook signing, secrets vault, port binding) is recommended but not implemented (9). The conceptual frame leans partly on non-peer-reviewed sources (a GitHub Gist and related commentary), which we flag as such (10). Latin-American accessibility is a motivation, not a demonstrated benefit; no Latin-American end-user was studied and non-English content was not tested.

### 4.8 Future directions

Priorities are: a benchmarked extraction-accuracy study against pre-stated ground truth on longer, multi-topic and deliberately ambiguous sessions; a fully self-hosted open-weights configuration with provider-neutral naming and self-hosted storage, evaluated for cost and dependence; a human verification gate and hardened pipeline (signed webhooks, secrets vault, network segmentation); multi-user and team-governance evaluation; memory compression for long projects; and, only with appropriate de-identification, governance and ethics approval, careful study of whether the pattern is useful adjacent to (never inside) clinical interpretation.

## 5 Conclusion

We describe and functionally demonstrate, as a single-user proof of concept, a transparent, file-based persistent-memory layer that carries project context automatically between a web conversational interface and a local coding agent for LLM-assisted radiology research and tool-development work. The system stores only development context and no patient data. Its contribution is an integration pattern and a user-owned, version-controlled memory artifact compatible with, and extending, the LLM Wiki pattern. We make no efficacy, accuracy or clinical claim; we are explicit about single-user scope, Claude-based implementation, text-only handling, absence of accuracy benchmarking, security work still to be done, and a cost model of fixed infrastructure plus variable API usage. For cost- and dependence-sensitive settings, including Latin America, we recommend a vendor-neutral open-weights configuration as the direction of further work, and we report that the system has already been useful in organizing the development of a separate radiology report quality-control tool.

## Data Availability

Replication resources are available at: https://github.com/angaritamd/ia-memory-radiology-resources. This repository contains the n8n workflow configuration (n8n-workflow.json), the Supabase database schema (supabase-schema.sql), the web-application API documentation (api-documentation.md), the extraction prompt template with example output JSON (prompt-template.md), templates of both memory files (templates/), and the Obsidian setup guide (obsidian-setup-guide.md). No patient data or clinical records were used. Further inquiries can be directed to the corresponding author.

## Appendix: Extraction prompt and model identifier

The following prompt was used for the Claude API extraction call (model: claude-sonnet-4, accessed April 2026 via the Anthropic Messages API v1/messages endpoint). The same prompt is provided in the replication repository (prompt-template.md). As noted in Section 2.4, the model identifier is not pinned to a minor version; any maintained deployment must be prepared to substitute the model identifier as providers update their model families.

**Table:**

Analyze this chat session between a user and an AI assistant. Project: {project} Date: {session_date} CHAT: {raw_chat} Respond ONLY with valid JSON, no markdown fences, no preamble: { “summary”: “one sentence describing the main achievement”, “decisions”: [“decision 1”], “pending”: [“pending task 1”], “artifacts”: [“artifact 1”], “errors”: [“error 1”], “continue_at”: “specific next step to resume” } Rules: decisions = choices made; pending = tasks left; artifacts = files produced; errors = problems (empty array if none); continue_at = one actionable sentence. Do not invent content not present in the chat.

The extraction model is swappable: the same prompt operates with self-hosted open-weights models (Llama-, Mistral- or Qwen-class) served behind an OpenAI-compatible endpoint, which is the recommended configuration where vendor dependence must be minimized (Section 4.3).
